# Intravenous Iron Infusion in the Treatment of Iron Deficiency Anaemia Following Bariatric and Metabolic Surgery and Correlation with Gynaecological Disorders: Retrospective Review of Experience from a Tertiary Centre

**DOI:** 10.3390/medicina61091647

**Published:** 2025-09-11

**Authors:** Emma MacVicar, Nivar Saleh, Joy Tneoh, James Lucocq, Georgios Geropoulos, Beverley Wallace, Anne Ewing, Peter J. Lamb, Gillian Drummond, Brian Joyce, Andrew G. Robertson

**Affiliations:** 1 General Surgery, Aberdeen Royal Infirmary, Foresterhill Health Campus, Aberdeen AB25 2ZN, UK; emma.macvicar2@nhs.scot; 2 Upper Gastro-Intestinal Surgical Unit, Royal Infirmary Edinburgh, 51 Little France Crescent, Edinburgh EH16 4SA, UK; nivar.saleh@nhs.scot (N.S.); joy.tneoh@nhs.scot (J.T.); james.lucocq@nhs.scot (J.L.); georgios.geropoulos@nhs.net (G.G.); beverley.wallace@nhs.scot (B.W.); peter.lamb@nhs.scot (P.J.L.); brian.joyce@nhs.scot (B.J.)

**Keywords:** bariatric surgery, iron levels, post-operative anaemia, weight-loss surgery

## Abstract

*Background and Objectives*: Iron deficiency anaemia (IDA) is a common consequence of bariatric and metabolic surgery (BMS). Women are at higher risk, and some patients cannot tolerate oral iron. This study aimed to report the demographics of patients with IDA that required iron infusion post-BMS and to investigate risk factors including gynaecological dysfunction. *Materials and Methods*: The medical records for all patients (*n* = 383) post-BMS at a large tertiary centre from January 2017 to December 2024 were reviewed, and those who received intravenous iron infusion (*n* = 32) for IDA were included. The criteria for iron infusion were ferritin < 50 µg/L or intolerance to oral iron. Demographic information including co-morbidities—gynaecological and other—age, pre-operative weight, body mass index (BMI), and ferritin levels were collected to investigate possible risk factors for IDA. *Results*: Thirty-two patients, all female, received one or more parenteral iron infusions. Eighteen had surgery locally; 14 had surgery elsewhere. Operations varied and included 14 Roux-en-Y gastric bypasses, 14 sleeve gastrectomy’s, and 4 gastric bands or other procedures. Eleven patients had a history of gynaecological disorders. Pre-infusion ferritin levels in the cohort with gynaecological disorders versus the cohort without were lower (median 11.0 vs. 14.5 µg/L), with a shorter time to presentation (median 6.9 vs. 10.2 years), and more patients requiring >2 infusions for resolution of symptoms (36.4% vs. 9.5%). *Conclusions*: Locally, 95% of our patients did not require iron infusion post-BMS. Eighty percent of those who did require iron infusion responded to ≤2 infusions. Women with a history of gynaecological disorder who underwent BMS required a significantly higher number of iron infusions and presented with symptoms sooner post-operatively vs. those without gynaecological disorders, particularly following Roux-en-Y gastric bypass. This is an important observation to consider both pre- and post-operatively for patients undergoing bariatric surgery, and additional well-designed studies that investigate this further are needed.

## 1. Introduction

Worldwide, almost half a million (449,815 primary procedures were recorded by the International Federation for Surgery for Obesity and Metabolic Management in 2023) bariatric and metabolic operations (elective weight-loss surgery) are performed annually. Most people who have bariatric surgery are female (3.9F:1M) with an average body mass index (BMI) of 40–45 kg/m^2^ at the time of surgery. The two most common primary operations performed are minimally invasive (laparoscopic and, more recently, robotic) sleeve gastrectomy (62.5%) and Roux-en-Y gastric bypass (28.5%) [1]. Both procedures have been shown to be safe and effective for weight loss, but patients may develop both short-term (within the first 30 days post-operatively) and long-term (>30 days post-operatively) complications. Patients undergoing bariatric and metabolic surgery (BMS) are also at risk of nutritional deficiencies [2].

Anaemia secondary to iron deficiency is a common long-term complication following bariatric surgery. People suffering with iron-deficient anaemia (IDA) may be asymptomatic, but more common presentations include fatigue, irritability, depression, difficulty concentrating, restless leg syndrome (32–40%), pica (40–50%), dyspnoea, light-headedness, and exercise intolerance [3].

Studies have shown that women in the general population are at higher risk of IDA than men: a haematology study published in the *Lancet* in 2021 found that, worldwide, the prevalence of IDA was 31.2% in females vs. 17.5% in males. One of the key reasons for this difference is menstruation and other gynaecological disorders [4,5].

Menstrual disorders make up approximately 12% of all referrals to gynaecology services in the United Kingdom (UK), with 1 in 20 women aged 30–49 years consulting their General Practitioner (GP) annually because of menorrhagia or menstrual problems [6].

Some of the other reasons for IDA post-BMS include pouch hypoacidity and a de-functioned small bowel. Other micronutrient deficiencies—zinc, copper, vitamins A and E, B12, and folate—may also contribute. Post-surgery human factors such as a change in taste and eating habits, non-adherence to recommended dietary intake, post-surgery food intolerance, e.g., being unable to tolerate red meat, and decreased food intake overall may also play a role. In addition, obesity is a state of chronic inflammation, and it is becoming increasing well known that this inflammation also plays a role in contributing to long-term post-BMS anaemia [7].

Polycystic ovarian syndrome (PCOS) is estimated to affect 6–13% of adult women of reproductive age and can cause irregular periods and heavy menstrual bleeding [6,8]. Uterine Leiomyoma (more commonly called uterine fibroids)—non-malignant tumours of smooth muscle cells and fibroblasts—are the most common benign pelvic tumour for women of reproductive age, affecting up to one in four members of the population. Approximately 30% of cases will cause heavy menstrual bleeding, which in some cases require hysterectomy for symptom management [9].

This study significantly adds to the literature by looking at the characteristics of all patients under review post-BMS over an 8-year period (January 2017–December 2024) who required parenteral iron supplementation at a large tertiary centre. Recent research has shown that women with gynaecological dysfunction are at increased risk of IDA post-BMS [8]; however, there is the need for further studies and investigations in this area, as research is limited. This is a particularly pertinent topic due to the large percentage of females undergoing BMS who may have gynaecological disorders, and the serious detriment nutritional and IDA may have on patients post-operatively. The primary aim of this study was to report the demographics of patients who develop IDA requiring iron infusion post-BMS. The secondary outcome was to determine risk factors for IDA in this group including gynaecological dysfunction.

## 2. Materials and Methods

**Figure 1:**
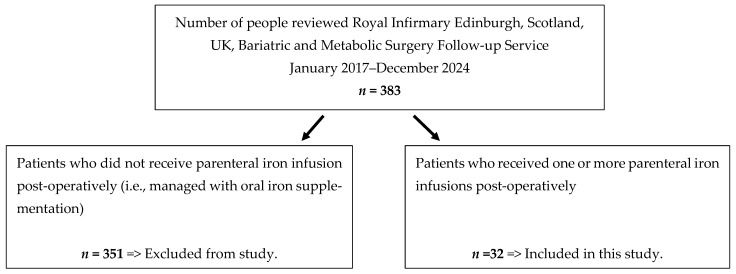
Summary of study population.

A retrospective review of a prospective database involving all patients from January 2017 to December 2024 who were referred to the specialist bariatric and metabolic service post-BMS at the Royal Infirmary of Edinburgh (RIE), a large tertiary centre in Scotland, United Kingdom (UK), was carried out (*n* = 383). All patients had BMS—either minimally invasive (laparoscopic or robotic) Roux-en-Y gastric bypass, sleeve gastrectomy or other operation (e.g., duodenal switch). Patients who received one or more iron infusions during this time period were identified (the patient selection process is summarised in Figure 1). The criteria for infusion of iron included ferritin < 50 µg/L despite oral iron therapy, or intolerance to oral iron.

### 2.1. Operative Technique

This has previously been described in the published literature [10,11]. To summarise—laparoscopic sleeve gastrectomy: the stomach was mobilised 5 cm from the pylorus to the left crus of the diaphragm. A 34 Fr gastric calibration tube was inserted, and the stomach stapled using a Purple 60 mm Endo-GIA (all staplers used are Medtronic Limited, Watford, UK) and Tan 60 mm Endo-GIA Stapler. A 2/0 Polydioxanone (PDS) suture was used to overrun the distal portion of the staple line.

Laparoscopic Roux-en-Y gastric bypass: the gastric pouch was created using an Endo-GIA 30 mm transverse and two vertical Tan 60 mm firings below the second branch of the left gastric artery. The biliopancreatic limb was measured to 50 cm and an antecolic, antegastric gastrojejunostomy was formed using a 45 mm Endo-GIA and the enterotomy was sutured. The Roux limb (a segment of the small intestine allowing food to travel from the gastric pouch to the small intestine, bypassing the duodenum) was measured to 150 cm and a jejunojejunostomy was formed with a 45 mm Endo-GIA and the enterotomy was sutured. The omega (duodenal–jejunal) limb was divided using a 60 mm Endo-GIA stapler. The jejunal–jejunal and Petersen’s defects were closed with an endohernia stapler.

### 2.2. Investigation of Iron Deficiency

Our unit protocol is to investigate all patients with IDA post-BMS who require an iron infusion. This is achieved via upper gastro-intestinal endoscopy, a quantitative faecal immunochemical test (qFIT), and 3D cross-sectional imaging via Computed Tomography (CT) of the patient’s chest, abdomen, and pelvis to rule out other causes (including colonic malignancy and upper gastro-intestinal ulceration) of anaemia prior to management of IDA with iron infusions. If qFIT is elevated >10 µg Hb/g then colonoscopy is performed.

### 2.3. Data Collection and Definitions

Data was then collected from electronic medical records by two independent investigators. This included sex, age at surgery, pre-operative weight and BMI, location of operation (i.e., UK, abroad), co-morbidities—both gynaecological (if applicable) and other conditions, pre-operative haemoglobin (Hb), mean cell volume (MCV), iron and ferritin levels, the number of iron infusions required, and time from surgery to first iron infusion. The haematology and biochemistry normal reference ranges were as follows—Hb: 115–165 g/L; MCV 78–98 fL; iron: 10–28 µmol/L; ferritin: 20–300 µg/L in post-menopausal women; and ferritin: 15–200 µg/L in pre-menopausal women [12].

Gynaecological co-morbidities included gynaecological referral to secondary care for menorrhagia (heavy bleeding), presence of uterine fibroids or ovarian cysts confirmed via imaging, and polycystic ovarian syndrome (PCOS). The diagnostic criteria for PCOS were as per National Institute of Health and Care Excellence (NICE) guidelines: to include two of the following: ovulatory dysfunction, clinical/biochemical signs of hyperandrogenism, and ovarian polycystic morphology on ultrasound (≥20 ovarian follicles in at least one ovary) [8].

Intravenous Ferinject^®^ (ferric carboxymaltose) was used for iron infusion, with dosing and administration carried out according to NICE guidelines and calculated based on the patient’s body weight and Hb (Appendix A). Ethical approval was provided via the NHS Lothian Health Board on 3 September 2024, and data was stored securely within the hospital server.

## 3. Results

Over a period of 8 years (January 2017–December 2024), a total of 383 people received post-operative BMS follow-up with our specialist BMS team. During this time period, 32 patients required parenteral iron infusion for IDA, and these patients were included in this study. Eighteen patients had their primary BMS performed locally in our unit and fourteen were referred from elsewhere due to refractory IDA. Initial operations were carried out between January 2007 and December 2023.

Patient demographics are summarised in Table 1. All patients were female (100%, *p* < 0.05), median age 36.5 years (range 23–55), with a median pre-operative weight of 111 kg (range 88.5–155) and a BMI of 46.1 kg/m^2^ (range 36.1–61.9). Overall, an equal number of patients underwent sleeve gastrectomy (14) and Roux-en-Y gastric bypass (14). Three others had gastric band placement and one had a duodenal switch operation (Table 1).

From the total cohort of 32, only 13 patients had no co-morbid conditions. 11 had a history of gynaecological disorder: 4 had menorrhagia, 1 had uterine fibroids, 3 had a diagnosis of PCOS, and a further 3 had known ovarian cysts. All 11 had these conditions at the time of surgery. Almost two-thirds (65.5%, 21) of people requiring iron infusion post-BMS had no known gynaecological disorder, and no other pathology (e.g., gastritis, malignancy) was found as a cause for their IDA. All patients are routinely prescribed oral iron lifelong post-BMS in our unit; therefore, the presumed underlying reason for deficiency was poor iron absorption due to their post-BMS anatomy or poor compliance with/intolerance to oral iron.

Fourteen patients had one or more other co-morbidities. These included fibromyalgia (3), type 2 diabetes mellitus (2), obstructive sleep apnoea (2), and hypertension (1). Only one patient had a haematological co-morbidity (platelet storage pool deficiency).

**Figure 2:**
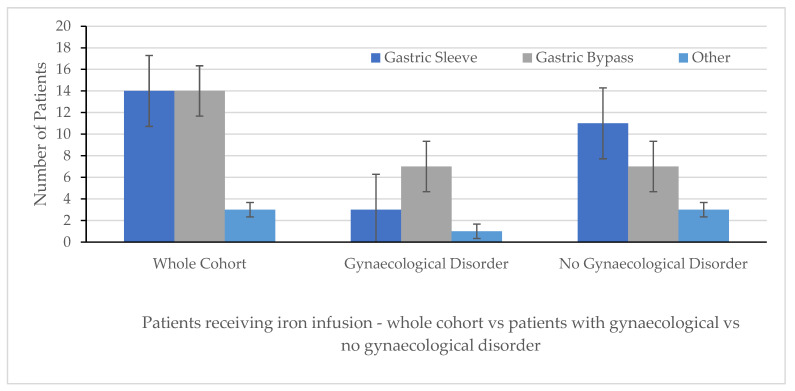
Comparison of operation types between patient cohorts: whole cohort (*n* = 32); those with gynaecological disorder (*n* = 11); and those with no gynaecological disorder (*n* = 21). The operations included, in order, are sleeve gastrectomy (labelled as gastric sleeve) vs. Roux-en-Y gastric bypass (labelled as gastric bypass) vs. other.

The group of patients with a history of gynaecological disorder (n = 11) vs. those without (n = 21) was then compared. Pre-operative ages and weights were similar between cohorts. There was a difference between operations: 63.6% (7/11) of patients with a gynaecological disorder requiring parenteral iron infusion had undergone a Roux-en-Y gastric bypass operation vs. 33.3% (7/21) of those without gynaecological disorders (Figure 2).

Pre-infusion median ferritin levels in the cohort with gynaecological dysfunction vs. the control cohort were lower (11.0 vs. 14.5 µg/L, respectively). The mean cell volume (MCV) pre-iron infusion was significantly lower for the gynaecological disorder cohort vs. the control cohort (84 vs. 88 fL *p* < 0.02). The median Hb (119 vs. 125 g/L) was also lower in the gynaecological dysfunction cohort, although the difference did not reach significance (*p* > 0.05) (Table 2).

**Figure 3:**
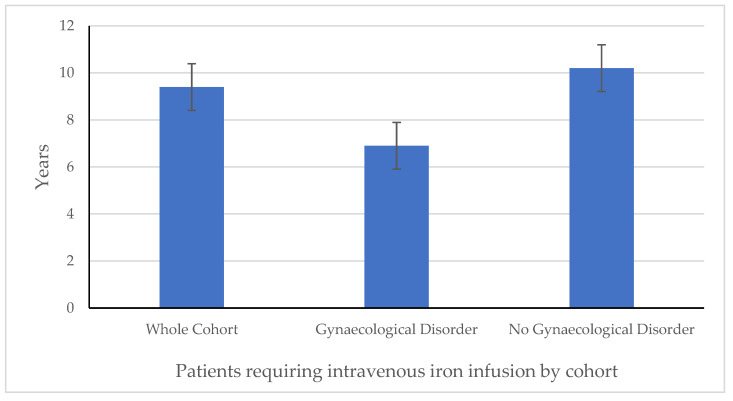
Time from surgery to first iron infusion in years: comparison between whole cohort, patients with gynaecological disorder (*n* = 11), and patients with no gynaecological disorder (*n* = 21).

There was also a difference in the time from surgery to the first iron infusion between the cohorts: overall, women who had a gynaecological disorder presented for iron infusion at an earlier time point post-BMS vs. those without any gynaecological disorder—a median of 6.9 vs. 10.2 years (Table 2 and Figure 3).

## 4. Discussion

The main finding of this retrospective analysis of a large prospective database is that around 5% of our local cohort of patients required iron infusions after BMS. All of the patients affected in our cohort were young females: we found that women are significantly more likely than men to require iron infusion post-BMS. Overall, around 80% of patients responded within two infusions: women with gynaecological disorders were more likely to require multiple (>2) infusions. In addition, women with gynaecological disorders presented for iron infusion with IDA at an earlier stage post-operatively vs. those without (6.9 years vs. 10.2 years).

The Increasing number of BMS operations being performed (449,815 primary operations recorded in 2023) and majority female patients (approximately 3.9 female patients for every 1 male) undergoing bariatric surgery worldwide makes this a highly relevant study. There is evidence in the literature that gynaecological dysfunction increases the risk of severe IDA requiring iron infusion post-BMS. Up to 1 in 20 women in the general population are affected by menstrual disorders, and this figure is likely to be higher in the bariatric population because of the effect of obesity and hyper-oestrogenism on gynaecological disorders and conditions like PCOS that can make obesity more likely. One study showed that pre-bariatric surgery, 38.6% of patients reported an irregular cycle, which then improved to 25.0% post-operatively [13].

A large study of 11,015 patients post-Roux-en-Y gastric bypass found an IDA rate of 16%. The Swedish Obesity Registry looked at anaemia post BMS in a cohort of 39,992 individuals—this study found a parenteral iron infusion requirement of 11% after 10 years. In addition, there was a significant difference in the number of women vs. men who were affected at 5 years post-bariatric surgery when 20.3% of women and 11.2% of men had been diagnosed with IDA. In the longer term, at 10 years post-operation, values were more equal between sexes, with 18.9% of women and 18.8% of men affected, i.e., almost one-fifth of patients were affected [14,15].

Body mass index (BMI) has been included in this study as part of the pre-operative assessment of our patient cohort. BMI is a contentious marker for assessing patients: as we move forward into the robotic age of bariatric surgery, we should consider using other, more representative measures to assess the success of bariatric surgery: kilograms lost or change in BMI does not give the full picture of the benefits of elective weight-loss surgery. It is important to highlight the need for change in practice and consideration of other factors in research studies—quality of life, mental health impact, the ability to participate fully in work and life, improvement in co-morbid conditions—e.g., a long-term reduction in the need for amputations due to diabetic foot complications and a reduction in hospital stays due to obesity-related morbidity are some of the essential markers of success that could be considered. In turn, quantifying and evidencing these factors (although clearly less straightforward to report versus calculating BMI) adds support to the argument that there needs to be further Investment and expansion in bariatric surgical provision, particularly considering the growing number of people with Class III obesity (BMI ≥ 40 kg/m^2^) worldwide. This is particularly relevant for the United Kingdom, where National Health Service budgets are limited.

It will also be interesting to see and review the impact of recently introduced weight-loss medications in the future: the literature shows there is a clear relationship between PCOS symptoms, infertility, elevated weight, and insulin resistance. If people undergoing bariatric surgery have a lower weight pre-operatively due to, for example, GLP-1 agonists, and are then able to maintain this over the long term with weight-loss surgery, we would expect the effects of their PCOS to be lessened: theoretically, this in turn may decrease the incidence of IDA requiring parenteral iron if there is a definite association between gynaecological dysfunction and IDA. This is a new and rapidly advancing area of medicine, and already it is likely we will see changes in medication use in the UK with the newly raised cost of these medicines from 2025 and ongoing limited availability under the NHS. The long-term impact of these medications on our patient cohort will be an important area for research in the future.

Another area for future study is carrying out qualitative interviews with the cohort of BMS patients who required parenteral iron infusion vs. those who did not and determining if, in retrospect, patients are happy with their decision to have BMS. Perhaps the benefits in their quality of life and improvements in co-morbid conditions due to weight-loss surgery far outweigh the inconvenience of having iron infusions (80% of our cohort responded to one or two infusions). Alternatively, it is feasible that patients may report that the need for infusions has had a significant impact on their life (one patient received 11 infusions during the study period), particularly given that it may be a lifelong problem unless they opt to undergo major revision surgery (reversal of Roux-en-Y gastric bypass for example). The authors have not found clear evidence in the literature to support either of these hypotheses; therefore, further qualitative work is needed to determine the effect of severe IDA on this patient group at a person level.

One positive finding of this study that is worth noting is that approximately 95% of patients operated on in our local area did not require post-operative iron infusion. This is important for surgeons counselling patients pre-operatively. For the 32 women who did require intravenous iron supplementation, most responded following one or two infusions. From our cohort, only six required four or more iron infusions: 66% (4/6) of these had Roux-en-Y gastric bypass surgery and 33% (2/6) had sleeve gastrectomy. It is interesting to note that all patients who underwent Roux-en-Y gastric bypass and later required more than the median number of iron infusions also had gynaecological disorders (fibroid, ovarian cysts, or menorrhagia). The two patients that had sleeve gastrectomy in this group had no gynaecological disorder. There is evidence from the literature that there is a higher rate of IDA and nutritional deficiency following gastric bypass vs. sleeve gastrectomy. However, in contrast to this, the By-Band-Sleeve study which has recently been published shows superiority of Roux-en-Y gastric bypass over sleeve gastrectomy for weight loss and quality of life [16]. Our results raise the following question: for women with known gynaecological disorder undergoing BMS, do we need to consider preferentially performing sleeve gastrectomy to decrease the risk of severe IDA post-operatively? With this small sample size and single-centre study, we cannot draw definite conclusions; however, it is an interesting question, and further multi-centre studies on a national and international level are warranted.

Recognising that patients with gynaecological abnormalities may be at increased risk of severe IDA requiring parenteral iron infusion post-operatively gives clinicians the opportunity to counsel at-risk patients appropriately pre-operatively and emphasise the importance of attending follow-up appointments with a specialist bariatric service. Careful patient selection is again highlighted to ensure that people opting to undergo BMS are willing and able to comply with the follow-up regime. This study also highlights the importance of the multi-disciplinary team—bariatric dietitians and medical specialists play an essential role, both pre- and post-operatively, in ensuring optimal care of patients by quickly identifying and correcting nutritional deficiencies, thus minimising morbidity from these conditions.

There is ongoing research at present regarding the optimal haemoglobin target for blood transfusion (for example, the “ABC post-ITU transfusion trial” based at the University of Edinburgh is a randomised controlled trial assessing the effect of red blood cell transfusion at a Hb of 100 g/L instead of 70–80 g/L, as is current practice). Our trigger target for iron infusion was ferritin < 50 µg: it would be interesting to further research this target and to consider if there is any benefit in prophylactically loading patients with iron, or whether we should consider earlier administration of parenteral iron post-operatively, particularly for those with a past medical history of gynaecological abnormality.

The most common non-gynaecological co-morbid condition from our cohort of patients was not one of the most obvious conditions associated with obesity—fibromyalgia. There is evidence from the literature that iron-deficient anaemia may not only make the symptoms of fibromyalgia worse but may also contribute to the development of the condition [17].

One limitation of this study is the small sample size of 32 patients: although differences were noted between cohorts, this may explain why they did not reach significance. In addition, we are restricted by the data available within medical records: body mass index was one area where data was incomplete, with 15/32 patients not having pre-operative BMI recorded or calculable, which may also have had a negative impact on our results and may have contributed to the non-significant *p* values. However, it is important to consider that little has been published on this topic, and our study provides an in-depth analysis of iron transfusion after bariatric surgery and its outcomes. Further studies exploring the factors that contribute to IDA requiring parenteral infusion post BMS in a larger population and with long-term follow-up are warranted.

## 5. Conclusions

Iron infusions are required in around 5% of patients locally after BMS. All of the patients in our cohort were young females and we found it was more common for females with gynaecological disorders to need a higher number of infusions and to present earlier post-operatively with IDA requiring infusion compared to patients without gynaecological co-morbidities. Around 80% of patients responded within two infusions. Female patients with gynaecological abnormalities may be at greater risk of more severe IDA post-BMS vs. women without such abnormalities. This study suggests that it may be helpful to identify this cohort of patients pre-operatively so that appropriate counselling regarding the risk of IDA and possible need for iron infusions in the long-term can be carried out. This study also suggests that for women with gynaecological disorders, Roux-en-Y gastric bypass operations may result in an increased risk of requiring repeated iron infusions post-operatively vs. sleeve gastrectomy: further well-designed, multi-centre studies are required to look at this question in more detail to allow us to draw definite conclusions before any recommendations to clinicians can be made. This study adds further evidence to the importance of all patients post-BMS receiving adequate supplements and monitoring to prevent morbidity from preventable IDA.

## Figures and Tables

**Table 1:** Summary of patient demographics of those requiring iron infusion post-BMS performed January 2017–December 2024 in a large tertiary centre (*n* = 32; total population *n* = 383).

	N = 32
Female/Male	32/0 (*p* value < 0.05)
Median Age, years (range)	36.5 (23–55)
Pre-operative weight, median, range (kg)	111 (88.5–155)
Pre-operative BMI, median, range (kg/m^2^)	46.1 (36.1–61.9)
**Location of Surgery:** Local UK other Europe USA Asia	18 (56.3%) 4 (12.5%) 8 (25.0%) 1 (3.1%) 1 (3.1%)
**Co-morbidities (gynaecological)** Uterine fibroid Menorrhagia Polycystic ovarian syndrome/Ovarian Cyst Nil	1 (3.1%) 4 (12.5%) 6 (18.8%) 21 (65.6%)
**Co-morbidities (non-gynaecological)** Fibromyalgia T2DM Obstructive sleep apnoea Hypertension (HTN) Haematological disorder Nil	3 (9.4%) 2 (6.3%) 2 (6.3%) 1 (3.1%) 1 (3.1%) 18 (56.3%)

**Table 2:** Summary of clinical data: comparison of values for all patients who required iron infusion (whole cohort, n = 32) vs. those with (*n* = 11) and without (*n* = 21) gynaecological co-morbidities. Haematology/biochemistry normal reference ranges: haemoglobin (female) 115–165 g/L, mean cell volume 78–98 fL; iron (female) 10–28 µmol/L. Ferritin: 20–300 µg/L in post-menopausal women; 15–200 µg/L in pre-menopausal women. Ns = non-significant [12].

	Whole Cohort (*n* = 32)	Gynaecological Disorder (*n* = 11)	No Gynaecological Disorder (*n* = 21)	*p* Values
Age median, (range) (years)	36.5 (23–55)	36 (26–48)	33.5 (23–55)	*p* = ns
Pre-operative weight (kg) median, range Pre-operative BMI (kg/m^2^) median, range	111 (88.5–155) 46.1 (36.1–61.9)	110.8 (93–133) 41.6 (39–43.8)	111 (88.5–155) 48.8 (36.1–61.9)	*p* = ns *p* = ns
**Pre-initial iron Infusion Values: median (range)** Hb (g/L) MCV (fL) Iron (µmol/L) Ferritin (µg/L)	123 (87–142) 87 (73–99) 10 (4–25) 13 (4–265)	119 (87–141) 84 (73–92) 13 (4–25) 11 (4–76)	125 (104–142) 88 (82–99) 9.5 (5–24) 14.5 (5–265)	*p* = ns *p* = 0.0119 *p* = ns *p* = ns
**Location of Primary Operation:** Home Institution UK Other Abroad	18 (54.3%) 4 (12.5%) 9 (28.1%)	5 (45.5%)06 (54.5%)	13 (61.9%) 4 (19.0%) 3 (14.3%)	
**Number of iron infusions administered** median, (range)	2.0 (1–11)	2.0 (1–11)	2.0 (1–5)	

**Figure A1:**
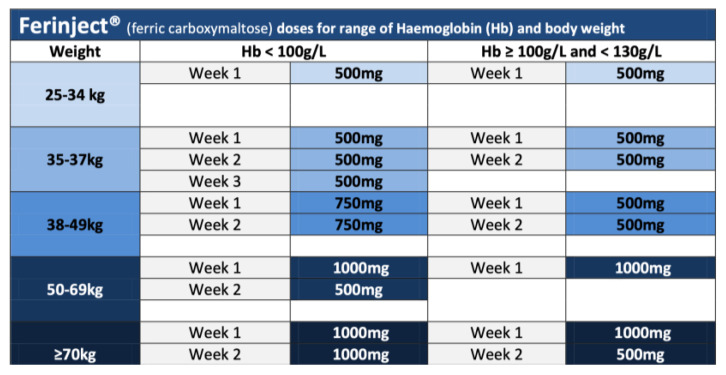
Ferinject^®^ dose calculation. Source: *Greater Glasgow and Clyde Medicines Handbook* [18].

## Data Availability

Dataset available on request from the authors.

## Abbreviations

The following abbreviations are used in this manuscript:

BMS	Bariatric and Metabolic Surgery.
IDA	Iron-deficient anaemia.
NICE	National Institute of Health and Care Excellence.
RIE	Royal Infirmary of Edinburgh.
qFIT	Quantitative Faecal Immunochemical Test.
USA	United States of America.
UK	United Kingdom.
PCOS	Polycystic ovarian syndrome.
NHS	National Health Service.

## Appendix A. Iron Infusion—Ferinject^®^—Dosage Calculation and Administration Regime

The cumulative dose for repletion of iron using Ferinject^®^ is determined based on the patient’s body weight and haemoglobin (Hb) level. If the BMI is more than 30 then the ideal body weight is used to calculate the dose.
